# Metabolic Concepts of Sodium-Glucose Cotransporter 2 Inhibitors-Based Therapies Against Hepatocarcinogenesis and Therapy Resistance in Hepatocellular Carcinoma

**DOI:** 10.3390/life16030446

**Published:** 2026-03-10

**Authors:** Hsien-Hui Chung

**Affiliations:** 1 Department of Pharmacy & Clinical Trial Pharmacy, Kaohsiung Veterans General Hospital, Kaohsiung City 813414, Taiwan; clinicaltrialchung@gmail.com; 2 Preventive Medicine Program, Center for General Education, Chung Yuan Christian University, Taoyuan City 320314, Taiwan; 3 Department of Pharmacy and Master Program, College of Pharmacy and Health Care, Tajen University, Pingtung County 907101, Taiwan

**Keywords:** anti-diabetic drugs, hepatocellular carcinoma, metabolic reprogramming, SGLT2 inhibitors, therapy resistance

## Abstract

The prevalence of hepatocellular carcinoma (HCC) has increased in recent years and resulted in many deaths, which necessitates new therapeutic solutions. The pathogenesis of HCC is associated with uncontrolled metabolic modulation and resistance to therapy. As diabetic carcinogenesis accelerates HCC progression, proper evaluation of anti-diabetic drugs to attenuate HCC is important. Although sodium-glucose cotransporter 2 (SGLT2) inhibitors that suppress renal SGLT2 are beneficial for treating diabetes, chronic kidney diseases, and heart failure, the use of SGLT2 inhibitors for treating HCC remains unclear. In this review article, some oncotargets involved in metabolic reprogramming, including glucose metabolism, Wnt/β-catenin, and hypoxia-inducible factor-1 alpha signaling, and the tumor microenvironment of HCC are briefly highlighted. Moreover, upregulated SGLT2 expression may be associated with hepatocarcinogenesis and therapy resistance, whereas the incorporation of SGLT2 inhibitors into combination therapies effectively attenuates HCC progression, metastasis, and therapy resistance through multiple mechanisms. Notably, how SGLT2 inhibitors modulate immune responses to cancer vaccines against HCC is highly appreciated and requires further evaluation. Thus, the clinical application of SGLT2 inhibitors in HCC and therapy resistance provides a promising direction for therapeutic strategies.

## 1. Introduction

The increasing trend in cancer-related mortality has been observed in hepatocellular carcinoma (HCC). HCC risk factors include aflatoxin exposure, hereditary hemochromatosis, primary biliary cholangitis, primary sclerosing cholangitis, hereditary hemochromatosis, autoimmune hepatitis, chronic hepatitis viral infection, alcohol-associated liver disease, and metabolic dysfunction-associated steatotic liver disease (MASLD) [1]. Clinical diagnosis of HCC is generally performed by using liver ultrasonography, multiphasic computed tomography, magnetic resonance imaging (MRI), liver biopsy, serum α-fetoprotein (AFP) levels, and reliable biomarker panels to detect tumor burden (vascular invasion, extrahepatic spread, and the number and size of nodules), and liver staging (very early stage, early stage, intermediate stage, advanced stage, and terminal stage) [2,3]. Surveillance, diagnosis, treatment, and post-treatment monitoring for HCC management are conducted in Taiwan with protein induced by vitamin K absence or antagonist II/des-gamma-carboxy prothrombin (PIVKA-II/DCP) and gadolinium-ethoxybenzyl-diethylenetriamine pentaacetic acid (Gd-EOB-DTPA)-enhanced MRI being employed for HCC detection, and antiangiogenic antibody (ramucirumab), oral multi-tyrosine kinase inhibitors (sorafenib, lenvatinib, regorafenib, and cabozantinib), and immune checkpoint inhibitors for monotherapy or combination therapy (pembrolizumab alone, atezolizumab in combination with bevacizumab, and ipilimumab in combination with nivolumab) being used for systemic HCC treatment [4]. According to the clinical practice guidelines of European Association for the Study of the Liver, the therapeutic strategies for HCC include invasive surgery, liver transplantation, radiation therapy, locoregional therapies (percutaneous ablation, intra-arterial embolization therapies, and external beam radiation therapy), chemotherapy, targeted therapy, and immunotherapy [5]. Currently, specific pathways in therapeutic patterns, such as (1) programmed cell death protein 1 (PD-1), programmed death-ligand 1 (PD-L1), and cytotoxic T-lymphocyte antigen 4 (CTLA4) linked to immune checkpoint pathways; (2) vascular endothelial growth factor receptor (VEGFR), transforming growth factor β (TGF-β) linked to angiogenesis-related pathways, and (3) mitogen-activated protein kinase (MAPK), phosphoinositide 3-kinase (PI3K)-protein kinase B (AKT), and Janus kinases (JAKs)/signal transducers and activators of transcription (STATs) linked to epigenetic modifications, play critical roles in HCC therapy [6]. Although clinical interventions for HCC have shown beneficial effects, therapeutic failure and drug resistance remain significant challenges. Therefore, the development of effective therapies for HCC is essential.

## 2. Metabolic Modulation Induced by SGLT2 Inhibitors in HCC

Liver cancer in Asian adults is closely associated with diabetes, with incidence and mortality rates of 5.3% and 5.8%, respectively [7]. Moreover, the pathogenesis of MASLD-related HCC is associated with hepatic lipid accumulation, oxidative stress, DNA damage, chronic inflammation and genomic instability in hepatocytes, and the survival and proliferation of HCC cells are enhanced by upregulated MAPK and PI3K-Akt-mammalian target of rapamycin (mTOR) pathways [8]. Thus, metabolic dysregulation of glucose, fatty acids, amino acids and glutamine metabolism contributes to the pathogenesis and progression of HCC linked to metabolic reprogramming, and the evaluation of anti-diabetic drugs for attenuating HCC is inevitable. Sodium-glucose cotransporter 2 (SGLT2) inhibitors, which block SGLT2 in the proximal tubules, are clinically prescribed to treat type 2 diabetes (T2D), heart failure, and chronic kidney diseases. In patients with MASLD and T2D, SGLT2 inhibitors exhibit lower risks of HCC and all-cause mortality than active comparators [9]. Compared to dipeptidyl peptidase 4 inhibitors, SGLT2 inhibitors have superior action in reducing the risk of liver cancers [10]. Moreover, the anti-cancer potentials of SGLT2 inhibitors have been thought to combat liver cancers via suppressed cancer cell proliferation and growth, and enhanced cell cycle arrest and apoptosis, which are related to systemic effects, as well as reduced β-catenin, glucose uptake, glycolysis, fatty acid synthesis, and oxidative phosphorylation, the induction of mitochondrial membrane instability, and increased 5’ adenosine monophosphate-activated protein kinase (AMPK) activity [11,12]. By using multi-omics analysis of metabolomics and absolute quantification proteomics in HCC cell lines (Hep3B and Huh7), SGLT2 co-localizes with mitochondrial pyruvate dehydrogenase kinase 1 involved in mitochondrial function, whereas canagliflozin suppresses HCC proliferation by activating AMPK through increased p-AMPKα1 and decreased p-AMPKα2 expressions, downregulating ATP synthase F1 subunit alpha related to mitochondrial oxidative phosphorylation metabolism, nucleoside diphosphate kinase 1 related to purine and pyrimidine metabolism, and upregulating 3-hydroxybutyrate and phosphorylation of acetyl-CoA carboxylase related to fatty acid metabolism compared to control [13]. Thus, the crucial pathways involved in the metabolic reprogramming of SGLT2 inhibitors against HCC are further summarized in Table 1.

### 2.1. Effects of SGLT2 Inhibitors on HCC via Glucose Metabolic Modulation

The shift from oxidative phosphorylation to aerobic glycolysis, which occurs during glucose metabolism in cancer cells, is known as the Warburg effect. It plays a critical role in the proliferation, invasion, and migration of liver cancer cells by increasing the glucose uptake to obtain more ATP. The expression levels of glucose transporter 1 (GLUT1) and GLUT12 in HCC are generally higher than those in healthy adjacent tissues, which are modulated by high glucose concentration, insulin, hormones, hypoxia, and acidic microenvironments whereas downregulated GLUT12 expressions exhibit anti-cancer properties, such as suppressed proliferation, invasion, and migration [14]. Moreover, higher expression levels of SGLT2 and GLUT1 along with related enzymes and substrates were observed in HCC, including pyruvate kinase M2 (PKM2) involved in glycolysis, fructose-1,6-bisphosphatase 1 (FBP1) and phosphoenolpyruvate carboxykinase 1 (PCK1) involved in gluconeogenesis, glucose-6-phosphate dehydrogenase (G6PD) and transketolase (TKT) involved in pentose phosphate pathway (PPP), and malic enzymes (MEs) and isocitrate dehydrogenase 2 (IDH2) involved in tricarboxylic acid cycle (TCA cycle), which are associated with cancer initiation, tumorigenesis, and progression [15]. Canagliflozin ameliorates the growth of SGLT2-expressing Huh7 and HepG2 cells by inhibiting glycolysis, inducing G2/M arrest via decreased extracellular signal-regulated kinase (ERK)1/2, p38, and AKT phosphorylation, enhancing apoptosis by increased caspase-3 cleavage, and suppressing intratumor vascularization in xenograft tumors [16]. Thus, SGLT2 inhibitors reduce HCC severity by targeting glucose metabolism.

### 2.2. Effects of SGLT2 Inhibitors on HCC via Wnt/β-Catenin Modulation

Wnt/β-catenin pathway plays a physiological role in regulating embryonic development, cell proliferation and differentiation. However, aberrant Wnt/β-catenin pathway enhances HCC development and progression, modulated via the crosstalk between conanical pathway and non-conanical pathway [17]. Liver tumor-initiating cells (TICs) have higher expressions of mitochondrial circRNA for translocating phosphoglycerate kinase 1 (mcPGK1) involved in self-renewal and metabolic reprogramming from oxidative phosphorylation to glycolysis by targeting PGK1, pyruvate dehydrogenase kinase 1 (PDK1), and pyruvate dehydrogenase (PDH) complex, which regulate Wnt/β-catenin activation by increasing lactate and decreasing α-ketoglutarate levels [18]. Moreover, canagliflozin inhibits HCC growth and increases survival both by disrupting glucose influx-induced β-catenin upregulation, and inhibiting protein phosphatase 2A (PP2A)-mediated β-catenin dephosphorylation [19]. Similarly, canagliflozin also alleviates hepatic fibrosis by reducing fibrotic markers’ (α-SMA and collagen I) expressions and attenuates the activation and proliferation of hepatic stellate cells (HSC-T6 and LX-2 cells) via PP2A/β-catenin signaling blockade [20]. Thus, SGLT2 inhibitors reduce HCC severity by targeting Wnt/β-catenin signaling.

### 2.3. Effects of SGLT2 Inhibitors on HCC via HIF-1α Modulation

Hypoxia-inducible factor 1 (HIF-1) is a dimeric protein consisting of an oxygen-sensitive subunit HIF-1α and a constitutively expressed subunit HIF-1β, and HIF-1α subunit exhibits physiological and pathological responses under hypoxia [21]. Regarding glucose metabolic reprogramming, the interaction between leucine–tyrosine–arginine motif containing 2 (LYRM2) and HIF-1α stability promotes HCC growth and metastasis by inhibiting mitochondrial respiration and increasing cellular glycolytic enzymes, such as hexokinase II (HK2), lactate dehydrogenase A (LDHA), PKM2 and GLUT1 [22]. Canagliflozin attenuates hypoxia-induced metastasis, epithelia-to-mesenchymal transition, angiogenesis, and metabolic reprogramming of HCC by targeting AKT/mTOR signaling to decrease HIF-1α protein accumulation [23]. Moreover, by activating AMPK and suppressing HIF-1α/Yes-associated protein (YAP)/transcriptional coactivator with a PDZ-binding motif (TAZ) pathway, canagliflozin significantly improves histopathological changes, liver function and oxidative stress; reduces antioxidant and stress response markers such as silent information regulator 1 (SIRT1) and nuclear factor erythroid 2-related factor 2 (Nrf2), inflammatory markers (STAT3 and P-STAT3) and tumor markers such as AFP, α-L-fucosidase (AFU) and carcinoembryonic antigen (CEA); elevates caspase-3 expressions; and decreases proliferating cell nuclear antigen (PCNA) expressions in a HCC rat model induced with choline-deficient diet (CDD) and diethyl nitrosamine and thioacetamide (DEN/TAA) [24]. Thus, SGLT2 inhibitors reduce HCC severity by targeting HIF-1α-mediated signaling.

**Table 1:** The metabolic modulation of SGLT2 inhibitors by multiple signaling pathways in HCC.

SGLT2is	Experimental Models	Main Outcomes	References
Canagliflozin	Hep3B and Huh7 cells	Canagliflozin inhibited the proliferation of HCC cells by modulating metabolic reprogramming of mitochondrial oxidative phosphorylation, fatty acid, and purine and pyrimidine metabolism	Nakano et al., 2020 [13]
Canagliflozin	HepG2, Huh7 cells, and xenograft tumors	Canagliflozin attenuated cell growth of SGLT2-expressing HCC cells by suppressing glycolysis, inducing G2/M arrest and apoptosis, and inhibiting intratumor vascularization in xenograft tumors	Kaji et al., 2018 [16]
Canagliflozin	Hep3B and Huh7 cells and xenograft tumors	Canagliflozin suppressed HCC growth and prolonged survival by disrupting glucose influx and inhibiting PP2A-mediated β-catenin dephosphorylation	Hung et al., 2019 [19]
Canagliflozin	HSC-T6 and LX-2 cells	Canagliflozin attenuated hepatic fibrosis by reducing fibrotic markers and inhibiting PP2A-mediated Wnt/β-catenin signaling in HSCs	Peng et al., 2025 [20]
Canagliflozin	HepG2 cells	Canagliflozin improved metastasis, angiogenesis, and metabolic reprogramming in HCC by targeting the AKT/mTOR pathway to suppress HIF-1α protein synthesis	Luo et al., 2021 [23]
Canagliflozin	CDD/DEN/TAA rats	Canagliflozin attenuated hepatocarcinogenesis in HCC-induced rats by AMPK activation and the inhibition of the HIF-1α/YAP/TAZ pathway	Fayed et al., 2025 [24]

CDD/DEN/TAA, choline-deficient diet/diethyl nitrosamine/thioacetamide; HSCs, hepatic stellate cells; PP2A, protein phosphatase 2A.

## 3. Mechanisms of SGLT2 Inhibitors in Therapy-Resistant HCC

Resistance to anti-cancer therapy in HCC is associated with metabolic reprogramming in the acidic and hypoxic tumor microenvironment (TME), and impaired anti-tumor functions of immune cells, such as T effector and natural killer (NK) cells [25]. Although upregulated SGLT2 expressions and increased glucose uptake were observed in cisplatin-resistant hepatoblastoma cells, combination treatment with dapagliflozin decreased glucose uptake and cisplatin resistance in resistant cells, and reduced tumor size in a xenograft model [26]. Moreover, the PI3K/AKT/glycogen synthase kinase-3β (GSK-3β)/mTOR and Wnt/β-catenin signaling pathways involved in γ-irradiation (γ-IR)-induced radioresistance could be overcome by prior treatment with canagliflozin in HCC, which is associated with inhibition of glucose uptake, lactate release, and the switch from modulation of endoplasmic reticulum (ER) stress-mediated autophagy to apoptosis induction [27]. Thus, SGLT2 overexpression is involved in both hepatocarcinogenesis and therapy resistance, which can be restored by SGLT2 inhibitors during metabolic reprogramming. Additionally, the oncogenic transcription factor c-Myc critically affects metabolic reprogramming of HCC via PI3K/Akt/mTOR, Wnt/β-catenin, and MAPK/ERK pathways, whereas the disruption of c-Myc-driven hepatocarcinogenesis by acetylation and ubiquitination ameliorates tumor proliferation, progression, and metastasis [28]. Regarding the role of c-Myc in therapy resistance, overexpression or amplification of c-Myc in HCC suppresses pro-inflammatory macrophages and is positively correlated with higher expressions of immune checkpoints PD-L1 and CTLA4, which limit therapy responsiveness and increase the risk of poor prognosis by reducing anti-tumor immune infiltration (Th1, Th17, CD4 T-cells, CD8 T-cells, NK cells, monocytes, and M1 macrophages). By contrast, combined anti-PDL1 and anti-CTLA4 therapy significantly attenuates c-Myc-driven HCC, restores macrophage-mediated anti-tumor immunity, and enhances antigen presentation by upregulating CD40 and major histocompatibility complex class II (MHCII) expressions without exerting severe toxicity [29]. However, the interactions between SGLT2 inhibitors and c-Myc in therapy-resistant HCC remain unclear. Notably, c-Myc overexpression upregulates SGLT2 expressions in HCC, and the treatment with empagliflozin attenuates c-Myc-mediated hepatic lipid accumulation, higher ATP content, cell proliferation and disease progression by directly inhibiting mTOR activation [30]. Moreover, independent of the original target SGLT2, canagliflozin increases HCC chemosensitivity to cisplatin by inhibiting glycolysis and modulating the PKM2-c-Myc complex and glutaminase 1 (GLS1)-mediated glutamine metabolism to induce ferroptosis [31]. Thus, multiple action mechanisms of SGLT2 inhibitors in therapy-resistant HCC are summarized in Table 2.

## 4. Combination Therapies with SGLT2 Inhibitors in HCC

The combination of SGLT2 inhibitors with chemotherapy or radiotherapy has been shown to enhance anti-cancer efficacy and improve therapy resistance [32], which can be considered as potent therapies for HCC. In a DEN-induced HCC mouse model, the combination of empagliflozin and metformin was superior to empagliflozin or metformin monotherapy in increasing survival, and reducing angiogenesis, invasion, progression, and metastasis via AMPK activation, autophagy activation, NF-κB inactivation, and the inhibition of MAPKs (p38 and ERK1/2) [33]. In human HCC cell lines (HepG2 and Huh7), combined treatment with canagliflozin and the dipeptidyl peptidase-4 inhibitor, teneligliptin, significantly suppressed cell proliferation compared to teneligliptin alone [34]. In non-alcoholic steatosis (NASH)-HCC progression, the use of combination therapy with tofogliflozin and the selective PPARα modulator, pemafibrate, significantly reduced tumor number and improved survival rates in a STAM mouse model via lipolysis induction and fatty acid re-esterification, and the inhibition of ER stress-related inositol requiring enzyme 1 α (IRE1α), X-box-binding protein 1 (XBP1), and pleckstrin homology-like domain family A member 3 (PHLDA3) pathway [35]. Moreover, compared to canagliflozin or sorafenib alone, the combination of canagliflozin and sorafenib significantly enhances HCC cell death in vitro and inhibits xenograft tumor growth in vivo by glucose starvation involved in glycolysis and the disruption of PTEN-induced putative kinase 1 (PINK1) and seven in absentia homolog 1 (SIAH1)-mediated mitophagy involved in mitochondrial respiration [36]. Thus, the beneficial effects of SGLT2 inhibitors-based combination therapies on HCC exhibit therapeutic potential in clinical patients, as shown in Table 3.

## 5. Putative Function of SGLT2 Inhibitors in HCC Cancer Vaccines

**Figure 1:**
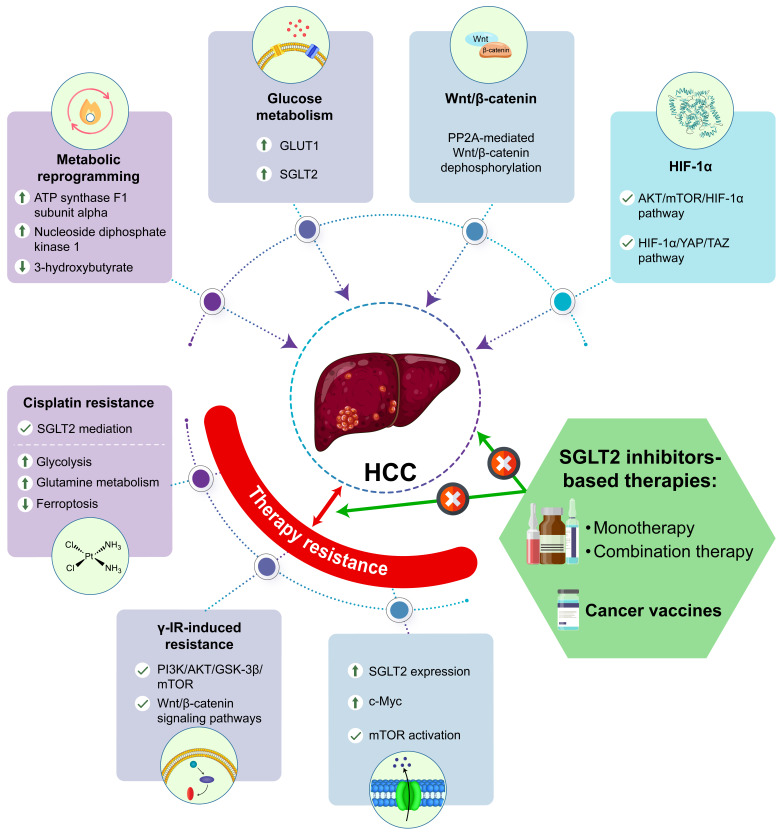
The metabolic modification of HCC and therapy resistance by SGLT2 inhibitors-based therapies. SGLT2 inhibitors-based therapies (monotherapy, combination therapy, and the adjuvant with cancer vaccines) attenuate both hepatocarcinogenesis-induced by metabolic reprogramming, aberrant glucose metabolism, Wnt/β-catenin, and HIF-1α-mediated pathways, and therapy resistance-induced by multiple metabolic carcinogenesis.

Although more promising therapies have been applied for HCC, the development of cancer vaccines as HCC therapies is emerging. Currently, immune responses to cancer vaccines are induced by cancer antigens and are manufactured using several biomedical materials, including RNA, DNA, peptide/protein antigens, viral antigens, viral or bacterial vectors, dendritic cells, red blood cells, embryonic material, whole tumor lysate, liposomes, exosomes, engineered bioinspired and biomimetic vaccines, and glycosylation-based vaccines. Moreover, the safety and efficacy of therapeutic cancer vaccines can be affected by critical factors, including disease complexity, adjustment of dosage, adjuvant and regimen of immunization, route of administration and delivery methods, identification of potent neoantigens, and intrinsic and extrinsic resistance mechanisms [37]. Therapeutic cancer vaccines not only eliminate established tumors but also reduce minimal residual disease and recurrence by activating and training the immune system [38,39]. However, some obstacles and challenges for developing personalized cancer vaccines must be overcome, including tumor visibility by the immune system, T cell suppression by the TME, T cell exclusion and tumor barriers, T cell exhaustion and dysfunction, large tumors, and metastatic cancer [40]. Notably, therapeutic cancer vaccines developed by screening suitable antigens from human HCC tissues and cell lines exhibit good safety and moderate immunogenicity [41]. HCC aggressiveness and postoperative recurrence are closely related to higher PD-L1 expression levels, and blocking the PD-1/PD-L1 pathway has synergistic effects with cancer vaccines in augmenting tumor antigen-specific T cell responses [42]. The combination of therapeutic cancer vaccines with radioembolization, chemoembolization, antiangiogenic drugs, and immunotherapy for HCC should be further considered and verified for therapeutic efficacy [43]. However, the role of anti-diabetic drugs as adjuvants in cancer vaccines against HCC remains unclear. Previous evidence has indicated that the downregulated expression of tumor PD-L1 by metformin partially through AMPK activation significantly enhanced tumor membrane vesicle (TMV) vaccine-induced inhibition of tumor growth and metastasis [44]. Moreover, SGLT2 expressions are positively correlated with PD-L1 expressions in patient lung cancer tissues, whereas canagliflozin ameliorates tumor progression by disrupting the interaction between PD-L1 and SGLT2, triggering the SPOP-mediated ubiquitin-proteasome degradation pathway during endocytic recycling, and increasing the antitumor activity of cytotoxic T cells [45]. Thus, SGLT2 inhibitors could possibly synergize with cancer vaccines to maximize the therapeutic efficacy and immune sensitivity through the metabolic modulation of TME and tumor-intrinsic mechanisms, providing novel directions for liver cancer therapy. In summary, the detailed clarification of direct or indirect metabolic crosslink in hepatocarcinogenesis of HCC (metabolic reprogramming, aberrant glucose metabolism, Wnt/β-catenin, and HIF-1α) and therapy resistance by SGLT2 inhibitors-based therapies should be further elucidated, as shown in Figure 1.

## 6. Safety, Pharmacokinetics, and Limitations of SGLT2 Inhibitors in HCC

The safety and pharmacokinetics of SGLT2 inhibitors in hepatic impairment in patients with HCC should be emphasized. Compared to the patients with cirrhosis receiving only furosemide and spironolactone alone, the combined use with SGLT2 inhibitors contributed to fewer paracenteses, hospitalizations, and serious liver-related events such as hyponatremia, ascites, variceal development, and all-cause mortality [46]. Moreover, the pharmacokinetics of SGLT2 inhibitors exhibits good tolerability, no new safety concerns and routine dose adjustment, mild adverse events, and the dependence of calculated creatinine clearance in different degrees of hepatic impairment [47,48]. Additionally, some limitations, such as off-target effects and heterogeneity among different SGLT2 inhibitors, are worthwhile investigating. Although SGLT2 inhibitors ameliorated HCC by directly or indirectly targeting multiple signaling pathways, off-target effects induced by SGLT2 inhibitors were reported and required careful monitoring, including genitourinary infections, volume depletion, and euglycemic diabetic ketoacidosis [49]. A meta-analysis of cohort studies has revealed significant heterogeneity and varying follow-up times for the use of SGLT2 inhibitors in reducing the risk of liver cancer [50]. Thus, the long-term evaluation of the safety, pharmacokinetics, and limitations of SGLT2 inhibitors for HCC treatment necessitates additional experiments and clinical trials.

## 7. Conclusions

This review summarizes the preclinical evidence and clinical outcomes regarding the metabolic benefits of SGLT2 inhibitors in ameliorating the carcinogenesis of HCC, facilitating the improvement of translational interpretation. Integrating multiple SGLT2 inhibitors-based therapies provides novel insights into more effective pharmacotherapies against HCC and its resistance to chemotherapy, radiation therapy, gene therapy, targeted therapy, immune therapy, and cancer vaccines. Applying artificial intelligence algorithms to HCC clinical features, current medications, biomedical data, genomics, transcriptomics, proteomics, metabolomics, biobanks, and clinical trials from diagnosis to prognosis can further enhance SGLT2 inhibitors-based therapies in attenuating HCC and improving the therapy resistance.

## Figures and Tables

**Table 2:** The resensitized mechanisms of SGLT2 inhibitors against therapy resistance in HCC.

SGLT2is	Experimental Models	Main Outcomes	References
Dapagliflozin	Cisplatin-resistant HepG2, HuH6 cells, and xenograft tumors	The reduced glucose uptake and cisplatin resistance in resistant cells, and decreased tumor size in xenograft model were mediated by SGLT2 inhibition	Fujiyoshi et al., 2024 [26]
Canagliflozin	γ-IR-induced radioresistance in HepG2 cells	The prior treatment with canagliflozin improved γ-IR-induced radioresistance in HCC related to reduced glucose uptake, lactate release, and induced apoptosis by PI3K/AKT/GSK-3β/mTOR and Wnt/β-catenin signaling pathways	Abdel-Rafei et al., 2021 [27]
Empagliflozin and Canagliflozin	HepG2 cell lines, transfected mice and human HCC tissues	The upregulated SGLT2 expressions were induced by overexpressed c-Myc in HCC, and c-Myc-mediated hepatic fat accumulation, cell proliferation and progression was improved by empagliflozin via directly suppressing mTOR activation	Rao et al., 2025 [30]
Canagliflozin	Cisplatin-resistant LM3, Huh7 cells and xenograft tumors	Canagliflozin reduced chemoresistance of HCC and induced ferroptosis by inhibiting glycolysis and targeting PKM2-c-Myc complex and GLS1-mediated glutamine metabolism	Zeng et al., 2023 [31]

γ-IR, γ-irradiation; GLS1, glutaminase 1; PKM2, pyruvate kinase M2 isoform.

**Table 3:** The effective anti-cancer action by combination therapies with SGLT2 inhibitors in HCC.

Combination Type	Experimental Models	Main Outcomes	References
Empagliflozin + Metformin	DEN-induced HCC mice	The increased survival, decreased HCC progression, anti-inflammation, and anti-oxidation were mediated by AMPK activation, NF-κB inactivation, and the inhibition of MAPKs, p38 and ERK1/2	Abdelhamid et al., 2022 [33]
Canagliflozin + Teneligliptin	HCC cell lines (HepG2 and Huh7)	The cell proliferation was significantly suppressed by combination therapy	Ozutsumi et al., 2020 [34]
Tofogliflozin + Pemafibrate	STAM mice	The significantly reduced tumor number and improved survival rates were mediated by inducing lipolysis and fatty acid re-esterification, and inhibiting the IRE1α-XBP1-PHLDA3 pathway	Murakami et al., 2022 [35]
Canagliflozin + Sorafenib	HCC cell lines (HepG2 and Huh7) and xenograft tumors	The promotion of HCC cell death in vitro and the inhibition of xenograft tumor growth in vivo were mediated by glucose restriction and disrupting PINK1 and SIAH1-mediated mitophagy	Zhou et al., 2022 [36]

DEN, diethyl nitrosamine; IRE1α, inositol requiring enzyme 1 α; PHLDA3, pleckstrin homology-like domain family A member 3; PINK1, PTEN-induced putative kinase 1; SIAH1, seven in absentia homolog 1; XBP1, X-box-binding protein 1.

## Data Availability

No new data were analyzed or created in this study, and data sharing was not applicable to this article.
